# A novel indication of platonin, a therapeutic immunomodulating medicine, on neuroprotection against ischemic stroke in mice

**DOI:** 10.1038/srep42277

**Published:** 2017-02-06

**Authors:** Joen-Rong Sheu, Zhih-Cherng Chen, Thanasekaran Jayakumar, Duen-Suey Chou, Ting-Lin Yen, Hsing-Ni Lee, Szu-Han Pan, Chih-Hsuan Hsia, Chih-Hao Yang, Cheng-Ying Hsieh

**Affiliations:** 1 Graduate Institute of Medical Sciences, College of Medicine, Taipei Medical University, Taipei, Taiwan; 2 Department of Pharmacology, College of Medicine, Taipei Medical University, Taipei, Taiwan; 3 Department of Cardiology, Chi-Mei Medical Center, Tainan City, Taiwan; 4 Department of Pharmacy, Chia Nan University of Pharmacy & Science, Tainan City, Taiwan

## Abstract

Thrombosis and stroke are major causes of disability and death worldwide. However, the regular antithrombotic drugs may have unsatisfactory results and side effects. Platonin, a cyanine photosensitizing dye, has been used to treat trauma, ulcers and some acute inflammation. Here, we explored the neuroprotective effects of platonin against middle cerebral artery occlusion (MCAO)-induced ischemic stroke in mice. Platonin(200 μg/kg) substantially reduced cerebral infarct volume, brain edema, neuronal cell death and neurological deficit scores, and improved the MCAO-reduced locomotor activity and rotarod performance. Platonin(5–10 μM) potently inhibited platelet aggregation and c-Jun NH2-terminal kinase (JNK) phosphorylation in collagen-activated platelets. The antiaggregation effect did not affect bleeding time but increased occlusion time in platonin(100 and 200 μg/kg)-treated mice. Platonin(2–10 μM) was potent in diminishing collagen- and Fenton reaction-induced ^∙^OH formation. Platonin(5–10 μM) also suppressed the expression of nitric oxide, inducible nitric oxide synthase, cyclooxygenase-2, interleukin-1β, and JNK phosphorylation in lipopolysaccharide-stimulated macrophages. MCAO-induced expression of 3-nitrotyrosine and Iba1 was apparently attenuated in platonin(200 μg/kg)-treated mice. In conclusion, platonin exhibited remarkable neuroprotective properties against MCAO-induced ischemia in a mouse model through its antiaggregation, antiinflammatory and antiradical properties. The observed therapeutic efficacy of platonin may consider being a novel medcine against ischemic stroke.

Stroke is the third leading cause of death and the most frequent cause of permanent disability worldwide^1^, and inflammation appears to be critical in the pathogenesis of ischemic stroke and other forms of ischemic brain injuries. The inflammatory response has a detrimental role in cerebral ischemia/reperfusion (I/R) injury pathogenesis^2^. The association between inflammation and cerebral I/R outcomes has ensured considerable and continued interest in the development of antiinflammation-oriented therapies for mitigating I/R-induced brain damage. In the brain, microglia and monocyte-derived macrophages are the key players in the immune response after stroke^3^; they are activated and migrate into the sites of injury following stroke. Microglia are rapidly activated upon brain injury and undergo substantial changes in morphology and functions, including proinflammatory protein production, and in behavior, including migration, proliferation, and phagocytosis^3^. By contrast, activated macrophages can switch to anaerobic metabolism and remain viable in hypoxic conditions. Therefore, hypoxic diseases including brain ischemia are correlated with macrophage activation^4^. The macrophages are activated by various inflammatory stimuli, including microbial lipopolysaccharide (LPS) and cytokines. As an inflammatory process progresses, macrophages excessively produce inflammatory mediators such as nitric oxide (NO), prostaglandin E2 (PGE2), and proinflammatory cytokines, including interleukin (IL)-1, IL-6, and tumor necrosis factor-alpha^5^. The subsequent generation of NO and PGE2 is catalyzed by inducible nitric oxide synthase (iNOS) and cyclooxygenase-2 (COX-2), respectively^6^. LPS initiates inflammatory cascades in macrophages through Toll-like receptor 4 (TLR4). Upon stimulation of TLR4, signaling pathways for the phosphorylation of Akt, c-Jun NH_2_-terminal kinase (JNK), extracellular signal regulated kinase (ERK1/2), and p38 mitogen-activated protein kinase (MAPK) are activated^7^. Thus, the inhibition of proinflammatory enzymes and cytokines is considered an effective therapy against neurodegenerative diseases.

A burst of reactive oxygen species (ROS) is produced during cerebral I/R, leading to the oxidation of lipids, proteins, and DNA and subsequently to cellular damage and apoptosis^8^. Therefore, much attention has been paid to the rescue of cerebral injury after I/R by inhibiting ROS bursts as a rational approach for preventing progression of neuronal damage during ischemic injury. Platelets are anuclear cells, critical to thrombus formation; after their activation by an agonist (such as collagen, ADP, and thrombin), platelets contribute to the amplification of the blood coagulation system^9^. Uncontrolled thrombus generation may lead to vascular disturbances and death. Thus, newer, safer, and more effective antithrombotic molecules, with no or few side effects, must be discovered or designed. Antiplatelet therapies, used for both managing and preventing ischemic stroke, reduce the incidence of stroke in patients at a high risk of thrombosis and in those with known symptomatic cerebrovascular disease. Although these therapies have some benefits, they have limitations, such as narrow therapeutic windows and indices, resulting in dietary or drug interactions; hence, they require monitoring and may produce serious side effects, including gastric disorders, bleeding, and thrombocytopenia^10^. Heparin and its analogs are included among such drugs associated with medication risks. Thus, alternative antithrombotic therapies are under extensive investigation, and many substances from natural sources are being isolated and studied to counteract the serious side effects^11^.

Platonin is a cyanine photosensitizing dye and a potent antioxidant. In addition to its free radical scavenging capacity, platonin has potent immunomodulating activity and is currently used for treating immune diseases in clinical settings^12^. We previously reported that platonin can alleviate circulatory failure and reduce mortality in septic rats^13^. Our data along with those from other studies^14^,^15^ confirm that platonin can attenuate endotoxin-induced inflammatory molecule upregulation. Although a recent study reported that platonin attenuates sepsis-induced loss of blood–brain barrier (BBB) integrity^16^, whether platonin exerts substantial protective effects in ischemic injury requires investigation. On the basis of the antiinflammatory activity of natural products in macrophages, which are closely related with brain damage, we hypothesized that platonin exerts antiinflammatory effects in macrophages and provides protection against brain injury, where macrophage-mediated inflammatory responses play a major pathogenic role. Because evidence indicates that antiplatelet therapy can prevent ischemic stroke in patients with several risk factors, this study also examined the *in vitro* antiplatelet effects of platonin.

## Results

### Platonin reduces infarct volume, brain edema and neuronal cell death in mice-subjected to MCAO

**Figure 1:**
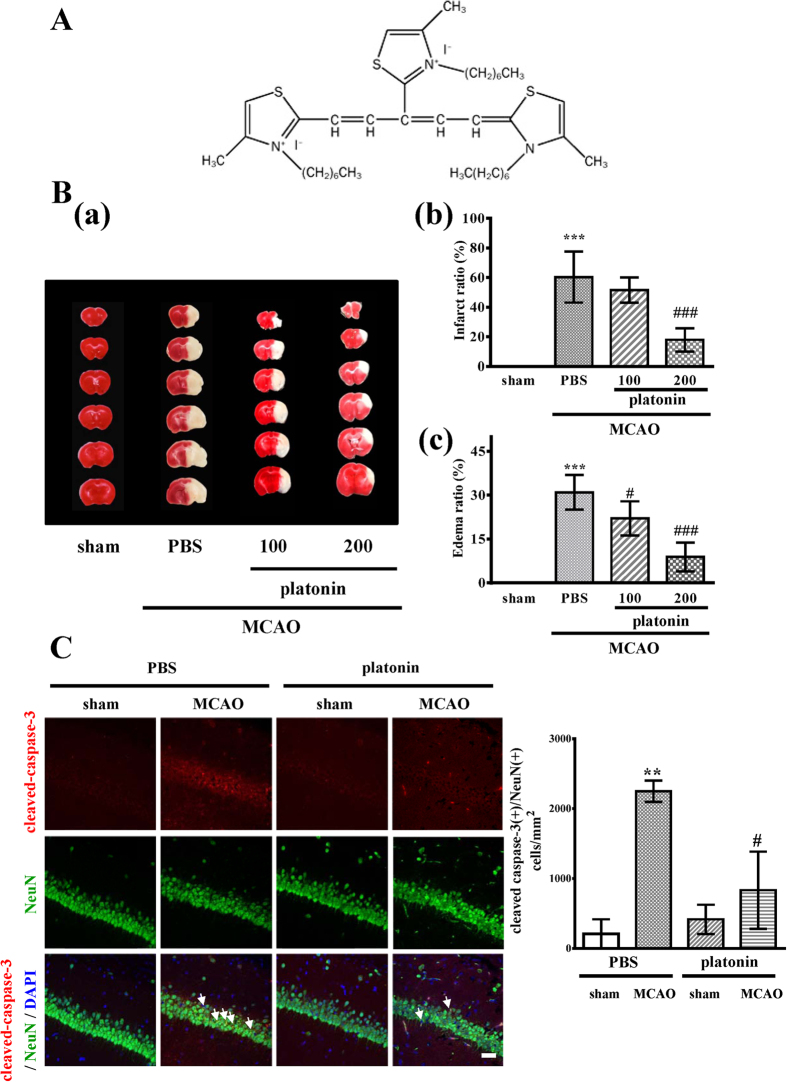
Protective effects of platonin against ischemic stroke in mice. (**A**) Chemical structure of platonin. (**B**) (a) Coronal sections of 2,3,5-triphenyltetrazolium chloride-stained brains after middle cerebral artery occlusion in the sham-operated group (sham), group treated with an isovolumetric solvent (phosphate-buffered saline (PBS), intraperitoneal [i.p.]), and groups treated with platonin (100 and 200 μg/kg, i.p.) for 30 min followed by embolic occlusion as described in Materials and Methods. The results represent eight similar experiments; densitometric analysis for the measurement of infarct volume (b) and brain edema (c) after treatment with platonin against embolic stroke in mice. Data are presented as the means ± SEM (n = 8). ***P < 0.001 compared with the sham group; ^#^*P* < 0.05, and ^###^*P* < 0.001, compared with the PBS group. (**C**) Mice were treated with the isovolumetric solvent control (phosphate-buffered saline (PBS), intraperitoneal [i.p.]) or platonin (200 μg/kg, i.p.) for 30 min and then subjected to middle cerebral artery occlusion (MCAO). The expression of cleaved-caspase-3 was determined at 24 h after surgery by using confocal microscopy as descripted in the Materials and Methods. Confocal images are typical of those obtained in three separate experiments demonstrating caspase-3 activation (arrows) in neurons of the ischemic portion of mice exposed to MCAO. Blue depicts the nucleus, red depicts cleaved caspase-3, and green depicts NeuN. The white bar indicates 40 μm. Data are presented as the means ± SEM (n = 3). **P < 0.01 compared with the solvent control (sham) group; ^#^*P* < 0.05, compared with the solvent control (MCAO) group.

The red region in the TTC-stained sections indicates the nonischemic portion of the brain, whereas the pale region indicates the ischemic portion. No infarction was observed in the sham-operated group, whereas extensive lesions developed in the striatum and lateral cortex in the MCAO group (Fig. 1B(a)). Treatment with 100 μg/kg platonin did not reduce the infarct volume; however, at a higher dose of 200 μg/kg, platonin significantly reduced the infarct volume compared with that in the solvent control group (*P* < 0.01) (Fig. 1B(b)). In addition, focal cerebral ischemia significantly increased cerebral edema in the ischemic hemisphere in the MCAO group. Pretreatment with 100 and 200 μg/kg platonin before MCAO resulted in a reduction of the brain edema increment (Fig. 1B(c)). The reduction of cerebral infarc volume and edema was also observed in MCAO mice treated with 400 μg/kg paltonin; however, there is no significant difference of therapeutic efficacy between the 200 and 400 μg/kg groups (data not shown).

By considering the important role of neuron in nervous system, the effect of platonin on neuronal cell death induced by ischemic stroke was also evaluated in this study. As shown in Fig 1C, the expression of cleaved-caspase-3 in neurons was obviously increased in mice exposed to MCAO (*P* < 0.01), and the pretreatment of platonin (200 μg/kg) potently attenuated cleaved-caspase-3 expression (*P* < 0.05).

### Platonin improves neurobehavioral functions

**Figure 2:**
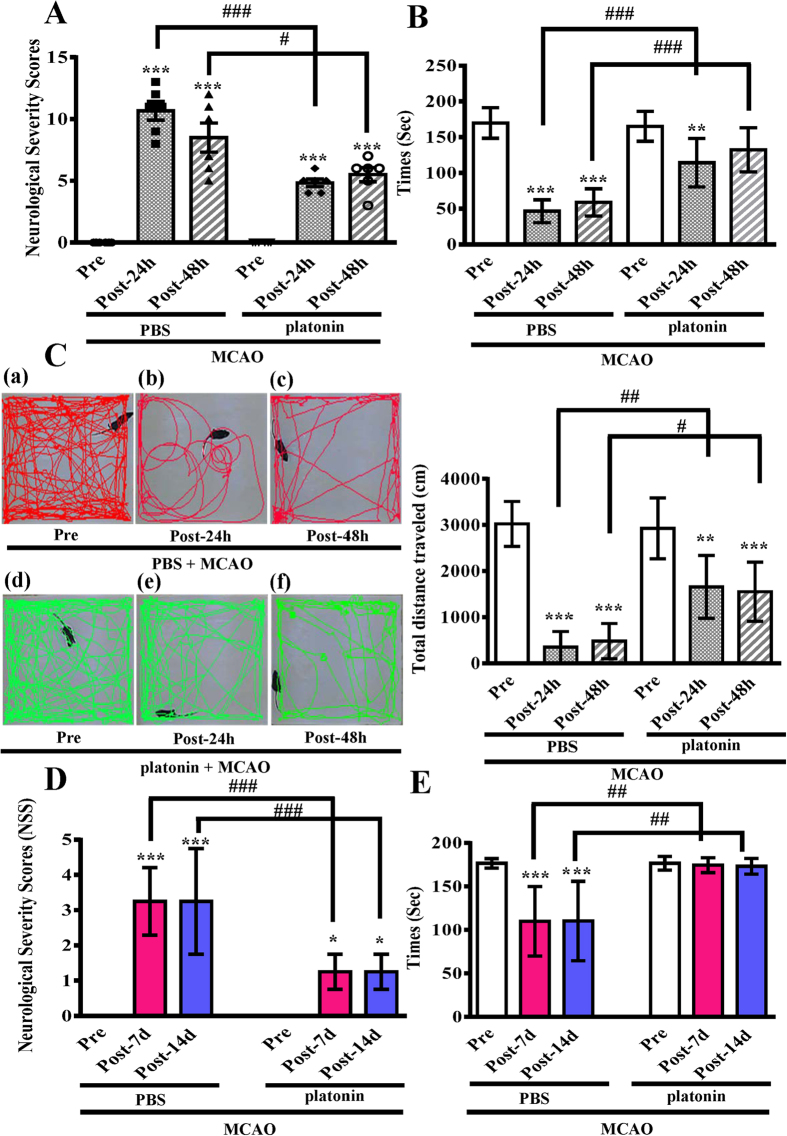
Alleviation of neurobehavioral deficits after platonin treatment in a mouse model of ischemic stroke. Mice were treated with the isovolumetric solvent control (phosphate-buffered saline (PBS), intraperitoneal [i.p.]) or platonin (200 μg/kg, i.p.) for 30 min and then subjected to middle cerebral artery occlusion. Assays of neurobehavioral functions including (**A**) neurological severity scoring, (**B**) the rotarod test, and (**C**) locomotor activity evaluation were performed before and 24 and 48 h after surgery. All data are presented as the means ± SEM (n = 6). **P < 0.01 and ***P < 0.001, compared with the presurgery group; ^#^*P* < 0.05, ^##^*P* < 0.01 and ^###^*P* < 0.001, compared with the PBS group. The therapeutic effects of platonin on long-term recovery were evaluated by (**D**) neurological severity scoring and (**E**) the rotarod test at 7 and 14 days after surgery. All data are presented as the means ± SEM (n = 3). *P < 0.001 and ***P < 0.001, compared with the presurgery group; ^##^*P* < 0.01 and ^###^*P* < 0.001, compared with the PBS group.

Neurological deficit was examined and scored on an 18-point scale before and 24 and 48 h after MCAO reperfusion injury. The changes in neurological deficit scores of different groups are illustrated in Fig. 2A. At 24 and 48 h after MCAO, neurobehavioral deficit scores significantly increased compared with those before MCAO; nevertheless, 200 μg/kg platonin significantly ameliorated these increased scores (Post-24 h groups: *P* < 0.001; Pre *vs.* Post-48 h groups: *P* < 0.05). The performance of the MCAO group mice in the rotarod test at 24 and 48 h after MCAO was significantly impaired; a decrease in the rotarod duration was prevented in the 200 μg/kg platonin-treated mice compared with the pre-MCAO group (Post-24 h groups: *P* < 0.001; Post-48 h groups: *P* < 0.001) (Fig. 2B). In addition, MCAO reperfusion injury significantly reduced locomotor activity (LMA) ((Pre *vs.* Post-24 h group: *P* < 0.001; Pre *vs.* Post-48 h group: *P* < 0.001); Fig. 2C(a–c)). Treatment with 200 μg/kg platonin extensively reversed the decrease in LMA occurring 24 and 48 h after MCAO injury (Post-24 h groups: *P* < 0.01; Post-48 h groups: *P* < 0.05; Fig. 2C(d–f)). Furthermore, the long-term recovery of mice exposed to MCAO was also evaluated in Fig. 2D,E. The neurological deficit and impaired rotarod performance of MCAO mice were significantly improved by the treatment of platonin (200 μg/kg) at 7 days and 14 days after surgery.

### Platonin affects vessel occlusion time, but not tail bleeding time, in mice

**Figure 3:**
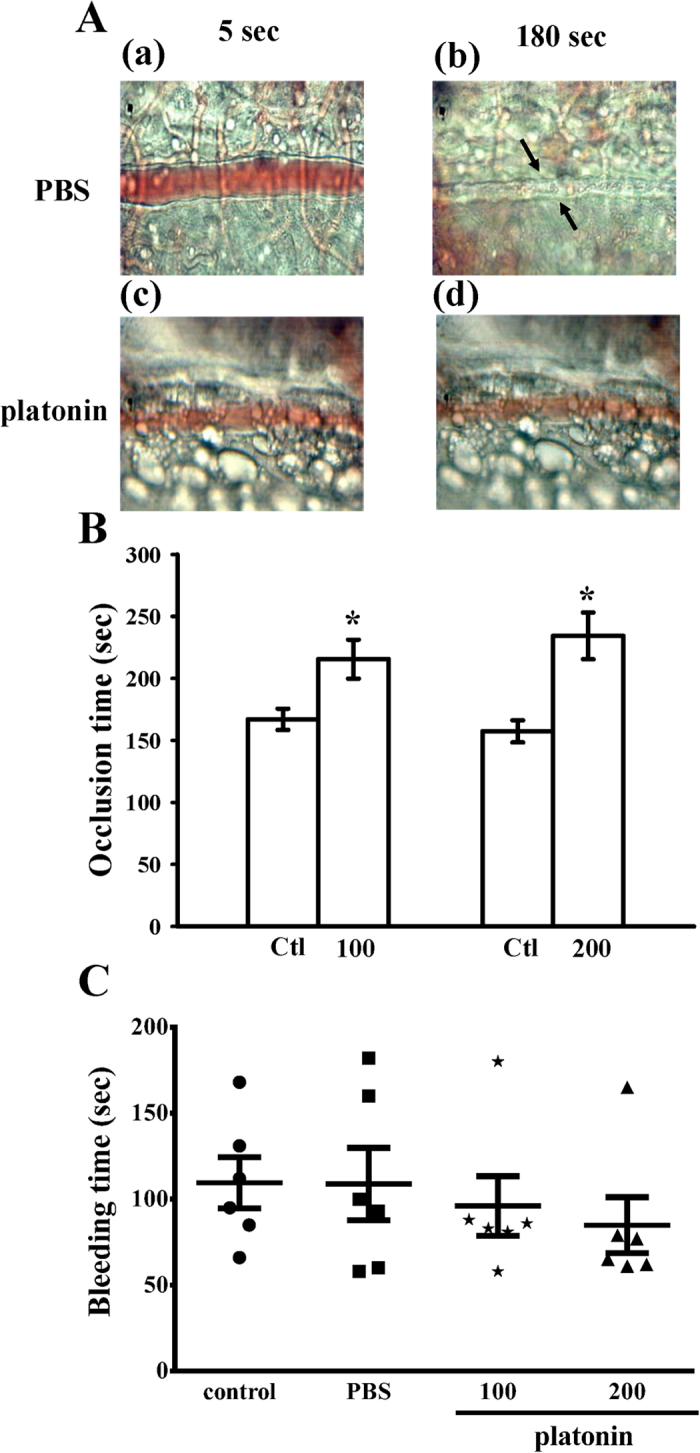
*In vivo* assay for the antithrombotic activity of platonin in the mouse models of thrombotic platelet plug formation and tail vein bleeding time. (**A**,**B**) Mice were treated with phosphate-buffered saline (PBS; isovolumetric control (Ctl), intravenous [i.v.]) or platonin (100 and 200 μg/kg, i.v.), and the mesenteric venules were then irradiated to induce microthrombus formation (occlusion time). (**A**) Microscopic images (400x magnification) of (a,b) PBS-treated controls and (c,d) the platonin (200 μg/kg)-treated groups were recorded 5 and 180 s after irradiation. Photographs represent six similar experiments. The arrows indicate platelet plug formation. Data are presented as the means ± SEM (n = 6). *P < 0.05, compared with the control group. (**C**) Bleeding time was measured 10 min after the intraperitoneal administration of PBS (isovolumetric control) or platonin (100 and 200 μg/kg) for 30 min. Data are presented as the means ± SEM (n = 6). Each symbol represents the bleeding time of an individual mouse.

The time to thrombotic occlusion in irradiated microvessels was approximately 180 s in the sodium fluorescein-pretreated mice; however, no platelet plug formation occurred at 5 s (Fig. 3A(a,b)). Moreover, 100 and 200 μg/kg platonin significantly prolonged the complete vessel occlusion time compared with that in the solvent-treated controls (*P* < 0.05) (Fig. 3B), as indicated by no platelet plug formation at 5 or 180 s (Fig. 3A(c,d)). By contrast, the same platonin dose did not affect the tail-vein bleeding time (Fig. 3C). These results indicated that platonin treatment, as an antiplatelet agent, did not elicit serious side effects on bleeding.

### Platonin inhibits collagen-induced platelet aggregation, free radical formation, and JNK phosphorylation in human platelets

**Figure 4:**
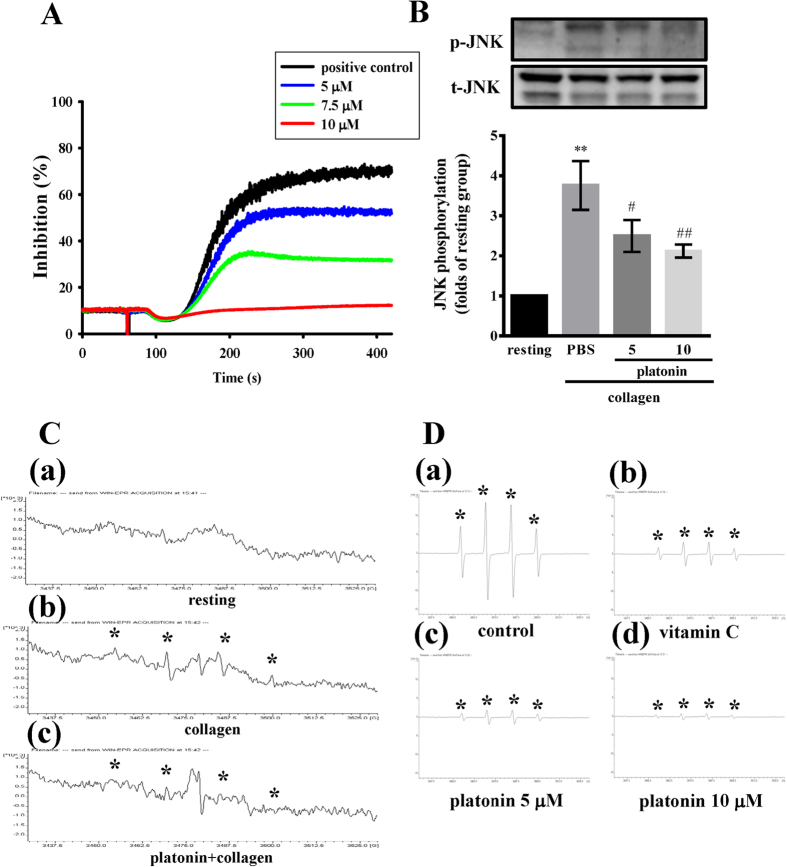
Suppression of aggregation, c-Jun NH_2_-terminal kinase (JNK) phosphorylation, and hydroxyl radical formation by platonin in collagen-activated platelets, and hydroxyl radicals formed from the Fenton reaction. (**A**) Washed platelets (3.6 × 10^8^/ml) were preincubated with phosphate-buffered saline (PBS; isovolumetric control) or platonin (5–10 μM) and subsequently treated with 1 μg/ml collagen to induce platelet aggregation (n = 3). (**B**) Washed platelets (1.2 × 10^9^ cells/ml) were preincubated with 5 or 10 μM platonin or PBS and subsequently treated with 1 μg/ml collagen to induce platelet activation. The platelets were collected, and subcellular extracts were analyzed to determine the degree of JNK phosphorylation. Data are presented as the means ± SEM (n = 3). **P < 0.01, compared with the resting group; ^#^*P* < 0.05 and ^##^*P* < 0.01, compared with the PBS group. For electron spin resonance analysis, (**C**) washed platelets (3.6 × 10^8^/ml) were (a) incubated only with Tyrode’s solution (resting group) or preincubated with (b) PBS or (c) 2 μM platonin and subsequently treated with 1 μg/ml collagen to induce hydroxyl radical (^**·**^OH) formation. (**D**) ^**·**^OH formed through the Fenton reaction was preincubated with (**a**) PBS (isovolumetric control), (b) vitamin C (100 μM), or (c,d) platonin (5 and 10 μM). Profiles C(a–c) and D(a–d) represent three independent experiments, and the asterisk (*) indicates ^**·**^OH formation.

To further understand the effect of platonin on platelet aggregation, we examined the influence of platonin on the aggregation of washed human platelets. Platonin showed inhibitory effects on collagen-induced platelet aggregation *in vitro* in a concentration-dependent manner (Fig. 4A). To identify the possible attenuating effects of platonin on platelets, we examined its influence on JNK phosphorylation and free radical formation in the platelets. As shown in Fig. 4B, collagen treatment significantly increased JNK phosphorylation (*P* < 0.01); this increment in JNK phosphorylation was reduced by 5 and 10 μM platonin in a concentration-dependent manner. Furthermore, 2 μM platonin reduced ^**·**^OH formation induced by 1 μg/ml collagen (Fig. 4C); in addition, ^**·**^OH produced through the cell-free Fenton reaction was scavenged by 5 and 10 μM platonin (Fig. 4D).

### Platonin diminishes LPS-stimulated proinflammatory mediators in RAW 264.7 cells

**Figure 5:**
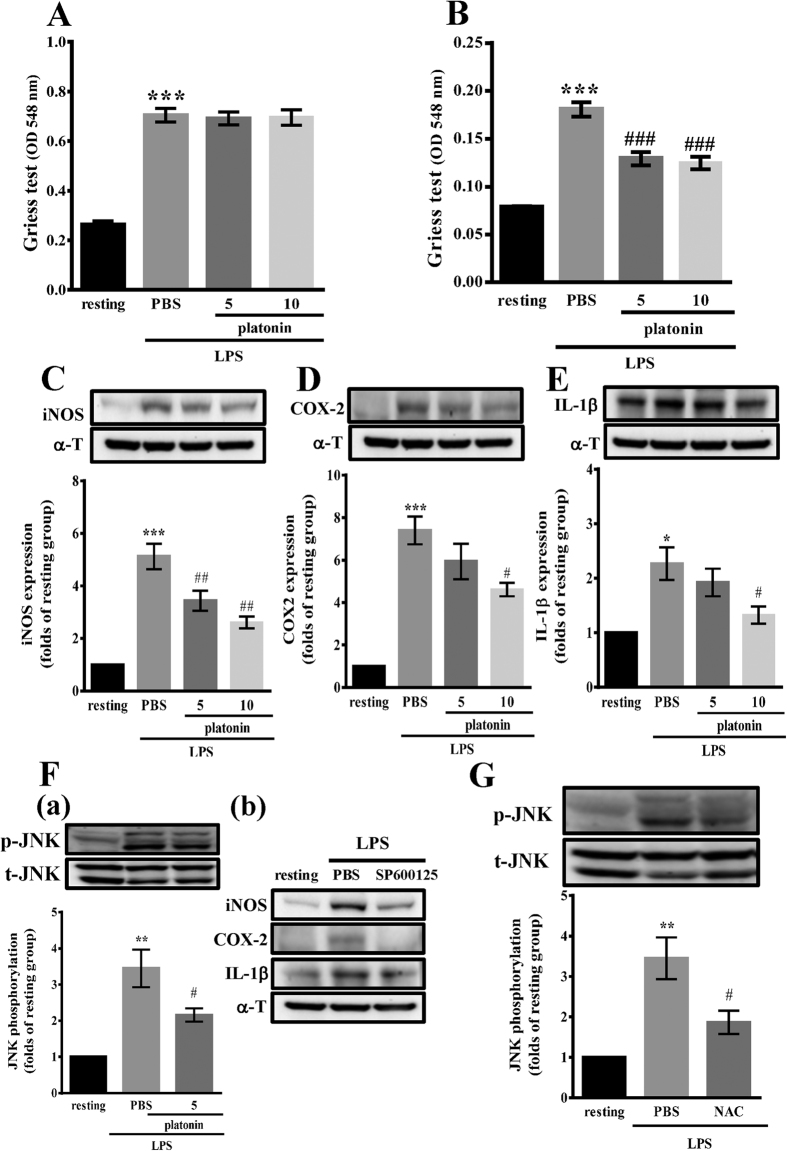
Antiinflammatory effects of platonin in lipopolysaccharide (LPS)-stimulated microglia and macrophages. (**A**) BV2 or (**B–G**) RAW 264.7 cells (8 × 10^5^ cells/6-cm dish) were treated without (resting) or with phosphate-buffered saline (PBS; isovolumetric control) or pretreated with platonin (5 and 10 μM), SP600125 (10 μM) or N-acetylcysteine (NAC) (10 mM) for 30 min and then treated with LPS (1 μg/ml) for 24 h (**A–E** and **F** (b)) or 30 min (**F** (a) and **G**). The nitrite concentration and inducible nitric oxide synthase (iNOS), cyclooxygenase-2 (COX-2), interleukin (IL)-1β and phosphorylated c-Jun NH_2_-terminal kinase levels were evaluated as described in Materials and Methods. Data are presented as the mean ± SEM (n = 3). *P < 0.05, **P < 0.01, and ***P < 0.001, compared with the resting group; ^#^*P* < 0.05, ^##^*P* < 0.01, and ^###^*P* < 0.001, compared with the PBS group.

To investigate the effect of platonin on LPS-stimulated NO production in BV2 and RAW 264.7 cells, nitric oxide production was evaluated by measuring the levels of nitrite released in the culture supernatants by using the Griess assay. As shown in Fig. 5A, a low NO level was observed in unstimulated BV2 cells. After LPS treatment, NO production considerably increased; however, 5 and 10 μM platonin did not suppress the increase in the NO levels in 1 μg/ml LPS-stimulated BV2 cells. By contrast, 5 and 10 μM platonin significantly inhibited NO production in LPS-stimulated RAW 264.7 cells in a concentration-dependent manner (Fig. 5B). Because NO is produced by iNOS, we next evaluated the effect of platonin on iNOS expression in LPS-stimulated RAW 264.7 cells. When cells were induced by LPS, iNOS expression considerably increased, and platonin treatment inhibited this expression in a concentration-dependent manner (Fig. 5C).

In addition to iNOS, the effects of platonin on the expression of COX-2 and IL-1β were evaluated in LPS-stimulated RAW 264.7 cells. In LPS-stimulated RAW 264.7 cells, COX-2 and IL-1β levels decreased after 5 and 10 μM platonin treatment (Fig. 5D,E). These data indicate potent antiinflammatory activity of platonin in activated macrophages.

### Platonin inhibits JNK phosphorylation in LPS-stimulated RAW 264.7 cells

We next assessed whether platonin inhibits LPS-induced JNK phosphorylation and whether the inhibitory effect of platonin on iNOS, COX-2, and IL-1β expression is dependent on the inhibition of JNK phosphorylation in RAW 264.7 cells. Pretreatment of cells with 5 μM platonin significantly reduced LPS-induced JNK phosphorylation (Fig. 5F(a)). Moreover, the LPS-induced expression of iNOS, COX-2, and IL-1β in RAW 264.7 cells was reversed by SP600125, a specific inhibitor of JNK (Fig. 5F(b)). This indicated that the inhibitory effect of platonin on iNOS, COX-2, and IL-1β may at least partially rely on inhibition of JNK phosphorylation.

The production of proinflammatory cytokines, ROS, and nitrogen species by macrophages represents the first line of defense; however, overproduction of these mediators can cause tissue and cell destruction^17^. Hence, ROS can mediate the activation of JNK^18^; we pretreated LPS-stimulated RAW 264.7 cells with N-acetylcysteine (NAC) to test whether the inhibitory effect of platonin on JNK phosphorylation is mediated through ^**·**^OH inhibition. The results showed that 10 mM NAC considerably diminished LPS-induced JNK phosphorylation in RAW 264.7 cells (Fig. 5G).

### *In vivo* antiinflammatory effect of platonin in mice exposed to MCAO

**Figure 6:**
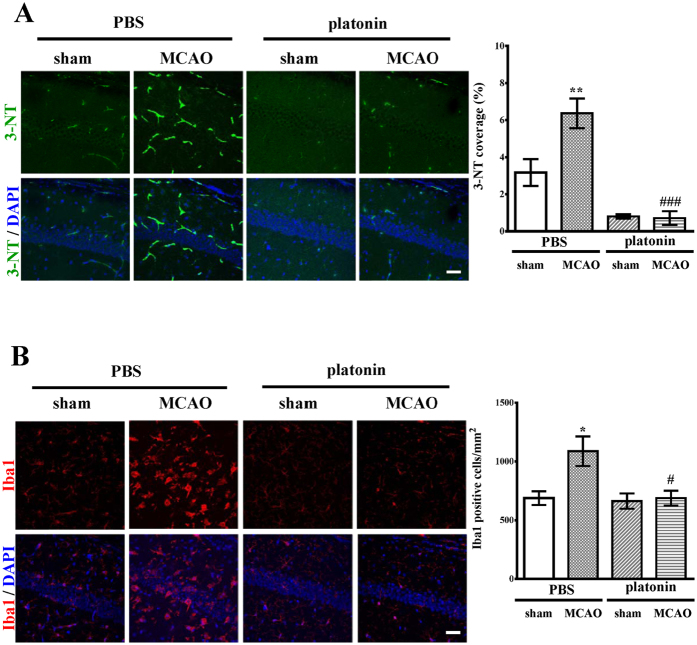
*In vivo* antiinflammatory effects of platonin in middle cerebral artery occlusion (MCAO)-treated mice. Mice were treated with the isovolumetric solvent control (phosphate-buffered saline (PBS), intraperitoneal [i.p.]) or platonin (200 μg/kg, i.p.) for 30 min and then subjected to MCAO. The brain slices were harvested 24 h after surgery. The expression of (**A**) 3-nitrotyrosine (3-NT) and (**B**) Iba1 were determined by using a confocal microscopic analysis as described in the Materials and Methods. Confocal images are typical of those obtained in three separate experiments demonstrating the expression of 3-NT and Iba1 in the ischemic portion of mice exposed to MCAO. Blue depicts the nucleus, green depicts 3-NT and red depicts Iba1. The white bar indicates 40 μm. Data are presented as the means ± SEM (n = 3). *P < 0.05 and **P < 0.01 compared with the solvent control (sham) group; ^#^*P* < 0.05 and ^###^*P* < 0.001, compared with the solvent control (MCAO) group.

Because of the potent inhibitory effect of NO production by platonin (Fig. 5), we further determined the *in vivo* antiinflammatory effects of platonin by evaluating 3-nitrotyrosine (3-NT), a biomarker of nitrogen free radical species modified proteins, in brain tissues. The expression of 3-NT apprently increased in the ischemic portion of mice-exposed to MCAO, and the pretreatment of 200 μg/kg platonin potently disminishing the expression of 3-NT (Fig. 6A) (P < 0.001). In addition, we also examined the expression of ionized calcium binding adaptor molecule 1 (Iba1), a specific marker of microglia/microphage, in the mice brain following MCAO. As shown in Fig. 6B, platonin (200 μg/kg) substantially attenuated the expression of Iba1 in mice exposed to MCAO. Iba1 positive cells were significantly decreased in the platonin-treated MCAO mice (*P* < 0.05).

## Discussion

The current study demonstrated the neuroprotective effects of platonin, a cyanine photosensitizing dye, on ischemia-induced brain damage by using a mouse MCAO model. This study also examined whether these neuroprotective effects are coupled with the inhibition of collagen-induced platelet aggregation in human platelets and LPS-induced inflammation in RAW 264.7 cells. The results revealed that platonin exerts neuroprotective effects by reducing the MCAO-induced infarct volume, brain edema and neuronal cell death as well as improving the neurological deficit score; in addition, it inhibits aggregation and inflammatory activities induced by collagen and LPS, respectively. Thus, the neuroprotective effects of platonin may be correlated with its antiaggregation and antiinflammatory activities. In general, the cerebral ischemia model with MCAO in experimental animals has been accepted as the most appropriate human stroke model^19^; thus, we used MCAO to induce ischemic injury in our mice.

Ischemia causes the release of cellular toxic mediators and increases the permeability of the BBB^20^. Hyperpermeability of the BBB leads to brain cellular swelling and causes brain infarction and edema^21^. A study proposed that the size of brain infarcts and edema are strongly correlated with neurological deficit severity^22^. In this study, MCAO caused significant brain infarction, edema, neuronal cell death and neurobehavioral dysfunction; however, platonin at a higher dose significantly inhibited these deficits. The more pronounced beneficial effect of platonin on LMA and motor coordination observed in MCAO-induced mice may be associated with a greater reduction in the lesion volume, as seen in this study. Therefore, the effects of platonin on LMA and rotarod performance in MCAO-induced mice were most likely due to its neuroprotection.

Because most strokes are caused by a thromboembolic occlusion of cerebral arteries, antithrombotic agents are the cornerstone of stroke prevention. Although the benefits of antiplatelet agents are uncertain in primary prevention^23^, they are still recommended for secondary prevention in patients who have experienced a noncardioembolic stroke^24^. However, commonly used antiplatelet agents such as cilostazol, clopidogrel, dipyridamole, and aspirin have various side effects including headache, internal bleeding, prolonged bleeding time, gastrointestinal bleeding, and palpitation^25^. Drugs that inhibit platelet activation and aggregation reduce vascular events, notably myocardial infarction and ischemic stroke, and are generally associated with low bleeding rates^26^. In this study, we observed that platonin inhibited collagen-induced human platelet aggregation. Increased risk of hemorrhage is a potential side effect of antithrombotic treatments^27^. To evaluate platonin in this aspect, we used the mouse tail transection model as an index of hemostasis. In this work, platonin did not alter the bleeding time appreciably, illustrating that platonin treatment for thrombosis is harmless, eliciting no side effects. In addition to platelet aggregation, the coagulation process is another determinant of the bleeding time and venous thrombosis occurrence^28^. To determine whether occlusion time is influenced by platonin, we evaluated the thromboembolic occlusion of cerebral arteries and observed that platonin dose-dependently prolonged occlusion time, an observation consistent with our previous study^29^.

In cerebral ischemic injury, inflammation is a crucial pathological process, contributing to neurovascular injury as well as affecting the progress of neurogenesis and brain repair. The poststroke inflammatory process involves multiple cells from resident inflammatory cells, such as microglia, to infiltrating blood-borne macrophages and neutrophils, which participate in the inflammation response^30^. Activated inflammatory cells secrete several inflammatory factors including cytokines, chemokines, enzymes, free radicals, and other small molecules, subsequently facilitating the inflammatory process and accelerating BBB breakdown and neuronal cell death or affecting brain repair^31^. Macrophages play vital roles in inflammation through the production of many proinflammatory molecules, including NO. NO overproduction can be harmful, resulting in various inflammatory diseases. Therefore, pharmacological interference with NO production may be useful in alleviating numerous disease states mediated by excessive and/or prolonged activation of macrophages. In the present study, we demonstrated that platonin inhibits LPS-induced macrophage activation and NO production in RAW 264.7 cells. Furthermore, we also directly observed a potent inhibition of MCAO-induced 3-NT and Iba1 expression in the brain of platonin-treated mice.

In addition to NO, macrophages also release prostaglandins, which are COX products. At least two genetically different COX isoforms—the constitutive COX-1 and the inducible COX-2—have been identified^32^. Here, IL-1β, iNOS, and COX-2 were induced after ischemia; these proinflammatory mediators contribute significantly to BBB breakdown, leading to edema, hemorrhage, and transmigration of leukocytes and large toxic molecules into the brain^33^. Accumulated data suggest that the MAPK signaling pathways positively regulate inflammatory molecule production; these pathways are upregulated after cerebral ischemia^34^. Studies have revealed that inhibition of the p38 and/or JNK MAPK pathways can directly or indirectly improve the outcome of ischemic brain injury by increasing neural cell survival through mechanisms including proinflammatory cytokine inhibition^35^. By contrast, we reported a critical role of JNK suppression in the inhibitory mechanism of platonin on vascular smooth muscle cell proliferation^36^. In this study, increased activation of phosphorylation of the proinflammatory enzymes COX-2 and iNOS and the proinflammatory cytokines IL-1β and JNK was observed in LPS-stimulated macrophages, and was significantly reduced by the platonin treatment. Furthermore, the JNK inhibitor SP600125 could reduce IL-1β, iNOS, and COX-2 secretion in LPS-stimulated macrophages. This finding partially corroborates previous results: pharmacological approaches with MAPK-specific inhibitors have demonstrated that the JNK pathway, but not the p38 and ERK pathways, is required for LPS-induced NO production and iNOS expression in RAW 264.7 cells^37^. Hence, here, the platonin treatment suppressed the MCAO-induced stroke by reducing LPS-induced inflammatory mediator levels and JNK phosphorylation, suggesting that platonin is a powerful antiinflammatory agent.

**Figure 7:**
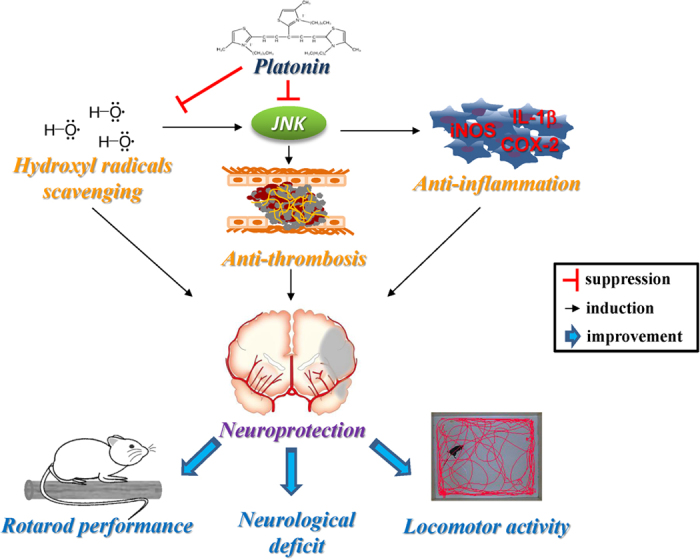
Diagram of the neuroprotective mechanism of platonin against ischemic stroke in mice. Platonin generates the neuroprotective effect against ischemic stroke through its anti-inflammation, anti-thrombosis, and hydroxyl radical scavenging properties. Consequently, neurological deficit, rotarod performance, and locomotor activity in a mice model of ischemic stroke were improved by the treatment of platonin.

Over the previous decade, major advances have led to a conclusion that ROS are generated and play a harmful role during cerebral ischemia-induced neuronal injury^38^. During ischemic injury, excessively generated ROS cannot be competently removed by the endogenous antioxidant systems, and the accumulation of ROS could cause oxidative damage to brain lipids, proteins, and nucleic acids, resulting in brain dysfunction and cell death^39^. High ROS levels are associated with high JNK levels in apoptotic cells^40^. By contrast, low ROS levels detected in the nearby surviving tissue are correlated with low, nondeleterious JNK levels and MAPK phosphatase activation. ROS may mediate JNK activation^20^ by quenching the MAPK phosphatases^41^. Thus, inhibition of JNK through ROS could protect the living cells close to those damaged from the noxious effects of high JNK levels. Our results showed that ^**·**^OH levels significantly increased in collagen-induced platelets and the Fenton reaction system and JNK phosphorylation increased in LPS-induced RAW 264.7 cells. Nevertheless, the platonin treatment significantly attenuated the increased ^**·**^OH levels as well as restored JNK phosphorylation. As an antioxidant, NAC can enhance the activity of tissue-specific antioxidant enzymes, such as SOD^42^. NAC can also inhibit NADPH oxidase activation, a source of ROS^43^. In general, NAC is used to identify the role of ROS in various biological responses. In the current study, pretreatment with NAC effectively reversed LPS-induced JNK phosphorylation in the RAW 264.7 cells, which indicated that ROS is critical for JNK activation. Therefore, the inhibitory effects of platonin on LPS and collagen-induced JNK phosphorylation may be associated with collagen- and Fenton reaction-induced ^**·**^OH inhibition. In conclusion, platonin has potential neuroprotective effects against MCAO-induced ischemic damage. We confirmed these results through an animal study, which demonstrated that platonin treatment significantly reduces infarct volume, brain edema, and neurological deficits 24 h after ischemic brain injury. The antiinflammatory, antithrombotic, and free radical scavenging effects of platonin may contribute to its neuroprotective potential in ischemic brain damage (Fig. 7). These results suggest that platonin may be a promising therapeutic drug for treating ischemic cerebrovascular diseases.

## Materials and Methods

### Materials

Platonin was synthesized by and obtained from Gwo Chyang Pharmaceuticals (Tainan, Taiwan; Fig. 1A). Dimethyl sulfoxide, bovine serum albumin (BSA), and SP600125 were purchased from Sigma-Aldrich (St. Louis, MO, USA). Dulbecco modified Eagle medium (DMEM), L-glutamine, penicillin, streptomycin, and fetal bovine serum (FBS) were purchased from Life Technology (Grand Island, NY, USA). Primary antibodies against cleaved-caspase-3, iNOS, COX-2, IL-1β, JNK, and phosphorylated JNK were purchased from Cell Signaling (Beverly, MA, USA). The anti-nitrotyrosine antibody was purchased from Santa Cruz (Santa Cruz, CA, USA). The anti-Iba1 antibody was purchased from Wako Chemicals USA (Richmond, VA, USA). The anti-NeuN antibody was from Merck Millipore (Darmstadt, Germany). The anti-α-tubulin monoclonal antibody was purchased from NeoMarkers (Fremont, CA, USA). Hybond-P polyvinylidene difluoride (PVDF) membranes, an enhanced chemiluminescence (ECL) Western blotting detection reagent and analysis system, horseradish peroxidase (HRP)-conjugated donkey antirabbit immunoglobulin (Ig) G, and sheep antimouse IgG were purchased from Amersham (Buckinghamshire, UK). For immunostaining, CF488A Donkey anti-mouse IgG, CF488A Donkey anti-rabbit IgG, CF594 Donkey anti-mouse IgG, and CF594 Donkey anti-rabbit IgG were purchased from Biotium (Fremont, CA, USA). Platonin was dissolved in phosphate-buffered saline (PBS) and stored at 4 °C.

### Animals

Male C57BL/6 mice (age, 6 weeks) were purchased from BioLASCO (Taipei, Taiwan). All animal experiments and care procedures were approved by the Institutional Animal Care and Use Committee (IACUC) of Taipei Medical University (TMU). Before undergoing the experimental procedures, all animals were clinically normal and free from apparent infection, inflammation, or neurological deficits.

### MCAO-induced cerebral ischemia in mice

The protocol conformed to the Guide for the Care and Use of Laboratory Animals (NIH publication no. 85–23, 1996), and was approved by IACUC of TMU, No. LAC-2015-0103. Male C57BL/6 mice were anesthetized using a mixture containing 75% air and 3% isoflurane maintained in 25% oxygen. The rectal temperature was maintained at 37 °C ± 0.5 °C. The right middle cerebral artery (MCA) was occluded as described in our previous study^44^. The right common carotid artery was exposed, and a 6–0 monofilament nylon thread (20 mm) coated with silicon (3 mm) was then inserted from the external to the internal carotid artery until the tip occluded the MCA origin. After closure of the operative site, anesthesia was withdrawn, and the mice regained consciousness. During another brief anesthesia period, the filament was gently removed after a 30-min MCAO. The mice were divided into four groups: (1) a sham-operated group; (2) a group treated with an isovolumetric solvent (PBS; intraperitoneal [i.p.]), followed by MCAO; and (3 and 4) groups treated with platonin (100 and 200 μg/kg i.p., respectively), followed by MCAO. All treatments were administered before MCAO in all the groups except the sham-operated group.

### Measurement of infarct volume and edema ratio

At 24 h after ischemic reperfusion, the mice were anesthetized; their brains were immediately removed, rinsed in cold saline solution, and sliced into 2-mm-thick coronal sections. The sections were immediately stained with 2% 2,3,5-triphenyltetrazolium chloride (TTC) for 30 min at 37 °C, followed by 4% formaldehyde solution overnight. The TTC-stained sections, with viable cerebral tissue stained red and infarcted cerebral tissue remaining pale, were photographed with a digital camera, and the infarcted areas of each section were measured using Image-Proplus (version 6.0) analyzer software. To compensate for edema formation in the ipsilateral hemisphere, infarct volumes were expressed as a percentage of the contralateral hemisphere volume as follows: infarct volume = (area of the intact contralateral left hemisphere) − (area of the intact ipsilateral right hemisphere)^44^. The edema ratio was calculated as follows: edema ratio = (ischemic volume − nonischemic volume)/(ischemic volume + nonischemic volume) × 100%. During the experimental periods of selected dose administration, no mortality was observed.

### Neurological severity examination

Neurological examination was performed on each mouse immediately before MCAO and at 24 h, 48 h, 7 days, and 14 days after injury^45^. Neurological Severity Scores (NSSs) were derived using an 18-point sliding scale (normal score, 0; maximal deficit score, 18). The NSS test includes motor, reflex, sensory, and balance tests. During the NSS test, 1 point represents the inability to perform the test or the lack of a tested reflex. Therefore, a higher score indicates severer injury.

### Spontaneous locomotor activity and rotarod assessments

Before neurobehavioral testing, the mice were trained daily for 3 days. They were trained to stay on an accelerating rotarod (4–40 rpm over 5 min, with increasing steps of 4 rpm at 30-s intervals) for three trials daily and subjected to the MCAO surgery. Before and 24 h after MCAO, the mice were placed in an activity monitor (Noldus Information Technology, Wageningen, Netherlands). Locomotor activity (LMA) was recorded automatically by counting the number of beam breaks in the test period. Total beam breaks were recorded in 10-min time bins over a 15-min period. After the LMA assessment, rotarod performance was assessed to test balance and coordination (UGO Basile, Varese, Italy). The rotarod was rotated from 4 to 40 rpm within 3 min. The time (in seconds) at which each mouse fell from the drum was recorded for up to 180 s by using a stopwatch.

### Immunofluorescent staining of brain tissues

Animals of each group were euthanized and perfused with 4% paraformaldehyde in phosphate buffer (PB). The brains were post-fixed with 4% paraformaldehyde in PB overnight. Before sectioning, the brains were immersed in 30% sucrose in PB for cryoprotection. The brains were sliced in the coronal plane at 50 mm and permeabilized with PB containing 0.2% Triton X-100 and 6% donkey serum for 60 minutes. These slices were incubated overnight at 4 °C with primary antibodies. Subsequently, the samples were washed 3 times with 0.2% tween 20 in PB and then exposed to secondary antibodies overnight. The prepared slices were then counterstained with DAPI (30 mM) and mounted using a mounting buffer (Vector Laboratories) on a glass coverslip. The samples were detected under a Leica TCS SP5 confocal spectral microscope imaging system using an argon or krypton laser (Mannheim, Germany).

### Fluorescein-induced platelet thrombi in mesenteric microvessels of mice

Our protocol conformed to the Guide for the Care and Use of Laboratory Animals (NIH publication no. 85–23, 1996), and was approved by IACUC of TMU, No. LAC-2015-0103. Thrombus formation was assessed as described in our previous study^46^. The mice were anesthetized, and an external jugular vein was cannulated with a PE-10 for administering the dye and drugs intravenously. Venules (30–40 mm) were selected for irradiation at wavelengths lower than 520 nm to produce a microthrombus. Platonin was administered at 100 and 200 μg/kg 1 min after the administration of sodium fluorescein (15 mg/kg); the time required to occlude the microvessel as a result of the thrombus formation (occlusion time) was recorded.

### Measurement of mouse tail vein bleeding time

This protocol conformed to the Guide for the Care and Use of Laboratory Animals (NIH publication no. 85–23, 1996), and was approved by IACUC of TMU, No. LAC-2015-0103. The bleeding time was measured through transection of the tail of the mice. After 30 min of 100 and 200 μg/kg i.p. platonin treatment, we sharply cut the tail of the mice at 3 mm from the tip. The tails were immediately placed into a tube filled with saline at 37 °C for measuring bleeding times. The bleeding time was recorded until the bleeding completely stopped for at least 10 min.

### Platelet aggregation

This study was approved by the Institutional Review Board of Taipei Medical University and conformed to the directives the Declaration of Helsinki. All human volunteers provided informed consent. Human platelet suspensions were prepared as described previously^46^. The blood was collected from healthy human volunteers, who had taken no medication during the preceding 2 weeks, and was mixed with an acid–citrate–dextrose solution. After centrifugation, the supernatant (i.e., platelet-rich plasma) was supplemented with 0.5 mM prostaglandin E1 and 6.4 IU/ml heparin. Washed platelets were finally suspended in Tyrode’s solution containing 3.5 mg/ml BSA. The final concentration of Ca^2+^ in Tyrode’s solution was 1 mM. A lumiaggregometer (Payton Associates, Scarborough, ON, Canada) was used to measure platelet aggregation, as described previously^19^. Platelet suspensions (3.6 × 10^8^ cells/ml) were preincubated with various concentrations of platonin (5–10 μM) or an isovolumetric solvent control (PBS) for 3 min before the addition of collagen (1 μg/ml). The reaction was allowed to proceed for 6 min, and the extent of aggregation was expressed in light-transmission units.

### Detection of hydroxyl radicals using electron spin resonance spectrometry

Electron spin resonance (ESR) spectrometry was performed using a Bruker EMX ESR spectrometer (Billerica, MA, USA), as described previously^47^. The platelet suspensions (3.6 × 10^8^ cells/ml) were treated with 1 μg/ml collagen and 2 μM platonin or PBS for 3 min in a separate vial. The suspensions were incubated for 5 min and 100 μM DMPO was added before the ESR analysis was conducted. The ESR spectrometer was operated at a 20-mW power, 9.78-GHz frequency, 100-G scan range, and receiver gain of 5 × 10^4^. Moreover, a Fenton reaction solution (50 μM FeSO_4_ and 2 mM H_2_O_2_) was pretreated with a solvent control (PBS) or platonin (5 and 10 μM) and vitamin C (100 μM) for 3 min.

### Cell culture and platonin treatment

The BV2 and RAW 264.7 cell lines were maintained in DMEM supplemented with 10% heat-inactivated FBS, penicillin G (100 units/ml), streptomycin (100 mg/ml), and L-glutamine (2 mM) and incubated at 37 °C in a humidified atmosphere containing 5% CO_2_. For platonin treatment, platonin was dissolved in PBS. The cells were pretreated with 5 or 10 μM platonin for 30 min and then stimulated with or without LPS (1 μg/ml) for the indicated time.

### Measurement of NO concentration

The nitrite concentration was determined using the Griess reagent (1% sulfanilamide and 0.1% naphthalenediamine in 2.5% phosphoric acid) as an indicator of NO production^48^. In brief, cells were plated at 8 × 10^5^ cells/well into a 6-cm dish in DMEM with 10% FBS. The cells were pretreated with platonin (5–10 μM) for 30 min and then stimulated with or without LPS (1 μg/ml) for 24 h. After incubation, 150 μl of the culture supernatants was mixed with 150 μl of the Griess reagent. The absorbance of the mixtures was measured at 550 nm on a microplate reader.

### Western blotting

To determine the level of COX-2, IL-1β, iNOS, and JNK phosphorylation in the whole cell lysate, protein samples were separated through 12% sodium dodecyl sulfate-polyacrylamide gel electrophoresis and transferred to a PVDF membrane by using a Bio-Rad semidry transfer unit (Hercules, CA, USA). The membranes were then blocked with TBST (10 mM Tris-base, 100 mM NaCl, and 0.01% Tween 20) containing 5% BSA for 1 h at room temperature and probed with the various specific primary antibodies. After several washes, the membranes were incubated with the HRP-linked secondary antibody (1:3000 in TBST) for 1 h. Immunoreactive bands were detected using an enhanced chemiluminescence system. The ratios of the semiquantitative results were obtained by scanning the reactive bands and quantifying the optical densities on a videodensitometer and Bio-profil Biolight software (version V2000.01; Vilber Lourmat, Marne-la-Vallée, France).

### Statistical analysis

Data are expressed as the means ± SEM, accompanied by the number of observations. The normality of the data was first tested using the Kolmogorov–Smirnov test. The continuous variables were compared using analysis of variance. When the analysis indicated significant differences among group means, each group was compared using the Newman–Keuls method. *P* < 0.05 was considered statistically significant.

## Additional Information

**How to cite this article**: Sheu, J.-R. *et al*. A novel indication of platonin, a therapeutic immunomodulating medicine, on neuroprotection against ischemic stroke in mice. *Sci. Rep.* **7**, 42277; doi: 10.1038/srep42277 (2017).

**Publisher's note:** Springer Nature remains neutral with regard to jurisdictional claims in published maps and institutional affiliations.
