# Author Correction: A novel indication of platonin, a therapeutic immunomodulating medicine, on neuroprotection against ischemic stroke in mice

**DOI:** 10.1038/s41598-020-62766-6

**Published:** 2020-04-06

**Authors:** Joen-Rong Sheu, Zhih-Cherng Chen, Thanasekaran Jayakumar, Duen-Suey Chou, Ting-Lin Yen, Hsing-Ni Lee, Szu-Han Pan, Chih-Hsuan Hsia, Chih-Hao Yang, Cheng-Ying Hsieh

**Affiliations:** 1 Graduate Institute of Medical Sciences, College of Medicine, Taipei Medical University, Taipei, Taiwan; 2 Department of Pharmacology, School of Medicine, College of Medicine, Taipei Medical University, Taipei, Taiwan; 3 Department of Cardiology, Chi-Mei Medical Center, Tainan City, Taiwan; 4 Department of Pharmacy, Chia Nan University of Pharmacy & Science, Tainan City, Taiwan

Correction to: *Scientific Reports* 10.1038/srep42277, published online 06 February 2017

This Article contains an error in Affiliation 2 which is incorrectly given as ‘Department of Pharmacology, College of Medicine, Taipei Medical University, Taipei, Taiwan’. The correct affiliation is given below:

Department of Pharmacology, School of Medicine, College of Medicine, Taipei Medical University, Taipei, Taiwan

